# Salinity Stiffens the Epidermal Cell Walls of Salt-Stressed Maize Leaves: Is the Epidermis Growth-Restricting?

**DOI:** 10.1371/journal.pone.0118406

**Published:** 2015-03-11

**Authors:** Christian Zörb, Karl H. Mühling, Ulrich Kutschera, Christoph-Martin Geilfus

**Affiliations:** 1 Institute of Crop Science, Quality of Plant Products, University of Hohenheim, Stuttgart, Germany; 2 Institute of Plant Nutrition and Soil Science, Christian-Albrechts-University Kiel, Kiel, Germany; 3 Institute of Biology, University of Kassel, Kassel, Germany; Huazhong Agricultural University, CHINA

## Abstract

As a result of salt (NaCl)-stress, sensitive varieties of maize (*Zea mays* L.) respond with a strong inhibition of organ growth. The reduction of leaf elongation investigated here has several causes, including a modification of the mechanical properties of the cell wall. Among the various tissues that form the leaf, the epidermis plays a special role in controlling organ growth, because it is thought to form a rigid outer leaf coat that can restrict elongation by interacting with the inner cell layers. This study was designed to determine whether growth-related changes in the leaf epidermis and its cell wall correspond to the overall reduction in cell expansion of maize leaves during an osmotic stress-phase induced by salt treatment. Two different maize varieties contrasting in their degree of salt resistance (i.e., the hybrids Lector vs. SR03) were compared in order to identify physiological features contributing to resistance towards salinity. Wall loosening-related parameters, such as the capacity of the epidermal cell wall to expand, β-expansin abundance and apoplastic pH values, were analysed. Our data demonstrate that, in the salt-tolerant maize hybrid which maintained leaf growth under salinity, the epidermal cell wall was more extensible under salt stress. This was associated with a shift of the epidermal apoplastic pH into a range more favourable for acid growth. The more sensitive hybrid that displayed a pronounced leaf growth-reduction was shown to have stiffer epidermal cell walls under stress. This may be attributable to the reduced abundance of cell wall-loosening β-expansin proteins following a high salinity-treatment in the nutrient solution (100 mM NaCl, 8 days). This study clearly documents that salt stress impairs epidermal wall-loosening in growth-reduced maize leaves.

## Introduction

Salinity worsens soil fertility, which is detrimental for the cultivation of most agricultural crop plants such as maize (*Zea mays* L.). Vegetative growth is reduced, which ultimately causes crop failures, even under low salt concentrations. The decline in biomass formation in response to a salt-induced osmotic-stress phase is caused by several factors including reduced leaf growth, because the smaller the photosynthetic organ, the lower the photosynthetic efficiency [1–5]. However, the mechanisms that cause stunned leaf growth during the osmotic stress-phase are not completely known [6, 7]. DeCosta et al. [8] observed, during their studies on barley and maize, that leaf and cell elongation rates are not limited by the magnitude of cell turgor under conditions of NaCl-induced osmotic stress. Instead, Cramer and Bowman [9] have suggested that the modified capacity of cell walls irreversibly to expand is a major growth-limiting factor that causes growth inhibition under the osmotic phase of salt stress. This hypothesis is supported by wall creep measurements that demonstrate reduced cell wall extensibility in expanding maize leaves during the first phase of salt stress, a phase that is dominated by negative osmotic effects [10].

Among the various tissues that build the leaf organ, the epidermis is thought to be of particular importance for controlling leaf growth. Since the epidermis possibly restricts the expansion of the leaf blade, Green [11] has postulated that the epidermis plays a decisive role with regard to the rate and direction of growth. In accordance, Kutschera [12] has concluded that the outer peripheral wall of the epidermis restricts the expansion of the entire organ, possibly by sending growth signals to the inner cell layers and because the outer wall of the epidermis is much thicker than the inner cell wall [13, 14]. Based on these physical properties, the epidermis is thought to resist the internal forces of turgor pressure generated by the underlying thin-walled tissues and, by this means, the epidermis might be able to regulate the rate of leaf growth. With respect to chemical signalling, Kutschera et al., [12, 15, 16] have reported that the epidermis is highly responsive to auxin, which enhances wall extensibility and cell elongation.

Based on the ideas proposed by Green [11] and Kutschera [12] who suggest a role of the epidermis in restricting the expansion growth of the leaf blade, we have designed this study in order to investigate whether the abiotic stress factor salinity impairs growth-related and cell-wall-loosening processes in leaf epidermal cells. To reveal contrasting physiological features and responses of the epidermal wall that might confer tolerance towards salinity, we have used maize plants that contrast in their degree of salt resistance. Growth-relevant parameters such as the capacity of the epidermal cell wall to expand, the abundance of growth-mediating wall-loosening β-expansin proteins and the growth-relevant apoplastic pH between the epidermal cells have been analysed in order to elucidate whether the epidermal layer, which is thought to control the expansion of the leaf blade, is particularly affected in the growth-reduced leaves of salt-stressed maize plants.

## Material and Methods

### Plant material and growth conditions

The salt-sensitive maize (*Zea mays* L.) hybrid Lector and the NaCl-resistant hybrid SR03 [17] were grown in hydroponic culture in a greenhouse. Over a period of 3 d, maize seeds were embedded in 1 mM CaSO_4_ (aerated solution at 25°C for 1 d) and placed between filter papers moistened with 1 mM CaSO_4_. The resulting seedlings were transferred to 4.5-L plastic pots (3 plants per pot) containing one-quarter-strength nutrient solution. After 2 d of cultivation, the concentration of nutrients was increased to half-strength and, after 4 d of cultivation, to full-strength for the prevention of osmotic stress. The nutrient solution was composed as described [18] as follows: 2.5 mM Ca(NO_3_)_2_, 1.0 mM K_2_SO_4_, 0.2 mM KH_2_PO_4_, 0.6 mM MgSO_4_, 5.0 mM CaCl_2_, 1 mM NaCl, 1.0 μM H_3_BO_4_, 2.0 μM MnSO_4_, 0.5 μM ZnSO_4_, 0.3 μM CuSO_4_, 0.005 μM (NH_4_)_6_Mo_7_O_24_, 200 μM Fe-EDTA. To avoid nutrient depletion, the solution was changed every 2^nd^ day. Salt (NaCl)-treatment started 2 days after the full-nutrient concentration was employed and was increased stepwise by 25-mM increments every 2^nd^ day to a final concentration of 100 mM (control plants were raised with 1 mM NaCl derived from the nutrient solution). Plants were harvested in the osmotic stress phase, 8 days after achieving the full salt stress treatment. The temperature was kept constant at 26°C during the light period and at 18°C during the dark period; relative humidity was ca. 70%. All measurements were based on expanding leaves that had started to develop at 1 day after the full 100 mM NaCl treatment was applied. Only leaves that exclusively grew for 8 days under 100 mM NaCl stress and corresponding controls were the subjects of this study. Regions of the youngest expanding leaf that emerged from the sheaths were investigated.

### Quantifying size of leaf epidermal cells

Epidermal cell size parameters, *viz*. length, width and surface area, were measured after the leaves had been embedded in a histological sectioning medium suitable for light microscopy. Cell size is referred to as “relative”, since measurements were obtained with the inherent spatial inaccuracy resulting from the embedding procedure[19]. Leaves were cut into small segments and immediately fixed as described elsewhere[20]. Thereafter, the leaf segments were dehydrated in a graded ethanol series and embedded in Technovit 7100 (Heraeus-Kulzer, Hanau, Germany). Subsequently, leaf-sections were cut with a microtome (10 μm). An inverted microscope (DMI6000B; Leica Microsystems, Wetzlar, Germany) set at 20-fold magnification was used for image collection. Measurements were carried out on corresponding images by using the calibrated scale bar provided by LAS AF software (Leica Microsystems, Wetzlar, Germany).

### Epidermal extensibility

Extensibility measurements of epidermal peels were carried out on isolated strips by using a linear variable differential transducer (LVDT) following the detailed procedure described by Hu et al. [21]. Frozen-thawed epidermal strips (length: 3 cm; width: 1.0 cm) were connected to the LVDT by a thread that was glued to the epidermal strip via a small clamp. The thread was gently tensioned to eliminate oscillations in LVDT output resulting from slipping and friction in the measurement system. Preliminary experiments indicated that this force did not affect leaf extension. Extension was induced by continuously increasing the counterweight by the addition of water at a flow rate of 0.1 mL per second into a container that was attached with a clamp and hook to the free end of the thread. Readings were taken from the transducer at second-by-second intervals. Time-course data were stored by a data logger (Delta-T Device, Cambridge, UK).

### Ratiometric *in planta* measurements of leaf apoplastic pH

Non-invasive apoplastic pH measurements were carried out on expanding leaves by using camera-based ratio analysis in combination with a pH-sensitive fluorophore. *Dye loading*. For the purpose of *in planta* measurements, the fluorescent pH indicator was loaded into the apoplast as described elsewhere [22]. Briefly, 25 μM Oregon Green 488-dextran conjugated to 10 kDa dextran (Invitrogen GmbH, Darmstadt, Germany) was directly inserted into the leaf apoplast by using a syringe. The 10 kDa-sized dextran guaranteed that the dye remained in the apoplast [23, 24]. *Inverse microscopy imaging*. An inverted microscope (DMI6000B; Leica Microsystems, Wetzlar, Germany) connected to a DFC-camera (DFC 360FX; Leica Microsystems, Wetzlar, Germany) via a 20-fold magnification, 0.4 numerical aperture, dry objective (HCX PL FLUOTAR L, Leica Microsystems) was used for image collection. An HXP lamp (HXP Short Arc Lamp; Osram, München, Germany) was used for illumination at excitation wavelengths of (ex) 440/20 nm and 495/10 nm. Excitation filters were switched by means of a filter wheel and exposure time was 150 mS for both channels. Dye fluorescence at both excitation channels was collected by using a 535/25-nm emission band-pass filter (BP 535/25; ET535/25M; Leica Microsystems) and a dichromatic mirror (LP518; dichroit T518DCXR BS, Leica Microsystems, Wetzlar, Germany). *Ratiometric analysis*. As a measure of pH, the fluorescence ratio F_495_/F_440_ was calculated on a pixel-by-pixel basis by using LAS AF software. Background noise values were subtracted at each channel. Quantitative measurements were calculated as the ratio of the mean intensity for user-defined regions of interest (ROIs). For converting fluorescence ratio data taken from living plants into apoplastic pH values, an *in situ* calibration procedure was performed as described elsewhere [25].

### Leaf surface protein extraction, Western blot and labelling of β-expansins

*Protein extraction*. Leaf surface proteins were extracted as previously described by Pyee et al. [26] by dipping the leaves in a mixture of chloroform: methanol (2:1) for 5 seconds. The leaf surface protein extract was then evaporated to dryness by rotary evaporation at 50°C under reduced pressure [27]. Proteins were suspended in protein sample buffer (5% v/v glycerol, 1.15% SDS, 2.5% β-mercaptoethanol, 0.125% bromephenol blue, 31.5 mM TRIS-HCl, at pH 6.8) and, subsequently, the protein concentration within the sample buffer was increased by using chloroform: methanol precipitation [28]. Proteins were resuspended in sample buffer. *1D PAGE*, *WB and immune-detection*. For Western blotting, 25 μg proteins were separated by 15% SDS-PAGE and were electro-blotted onto a PVDF membrane (BioTrace, PALL Life Sciences, Pensacola, USA) by using a semi-dry transfer system (SemiPhor, Hoefer Pharmacia Biotech, San Francisco, USA). Briefly, SDS gels and the PVDF membrane were equilibrated for 15 min in Towbin buffer (25 mM TRIS-base; 192 mM glycine; 20% v/v methanol; 0.01% w/v SDS; pH 8.3) and subsequently placed between soaked sheets of filter paper (Whatman gelblot paper; 1.2 mm, Sigma, Munich, Germany). Transfer was conducted for 75 min at 40 V, 0.8 mA/cm^2^. Efficiency was checked with the pre-stained protein marker. *Immuno-detection*. For immune-staining, the membrane was saturated in a solution of milk powder in TRIS-buffered saline (TBS) with Tween (TBS-T; 2.5% w/v bovine skimmed milk; 0.1% v/v Tween) for 2 h at room temperature and then incubated with an anti-peptide rabbit IgG antibody (anti-ZmExpB). The antibody was raised against a conserved 15-amino-acid region shared by seven different, vegetatively expressed, β-expansin isoforms [29] (purchased from BioTrend; Cologne, Germany). The blot was incubated with the antiserum diluted 1/250 in the same TBS-T solution (0.1 mL/cm^2^ PVDF membrane) for 2 h and washed three times with TBS-T (0.1% v/v). Subsequently, the membrane was incubated with horseradish-peroxidase-conjugated goat anti-rabbit IgG (Sigma-Aldrich, Munich, Germany), diluted 1/38000 in TBS-T, for 1 h at room temperature. Finally, the membrane was washed three times in TBS-T and once in TBS. Signals were detected by using chemi-luminescent peroxidase subtrate-3 on Kodak BioMax light film (Sigma, Munich, Germany) with an exposure time of 5 minutes. Negative controls with pre-immune serum did not reveal any specific bands (data not shown).

### Specificity of immune-chemical β-expansin labelling

*Principle*. Before the anti-peptide antibody (anti-ZmExpB) was used for the detection of β-expansin by Western blotting, its specificity was tested following the method of Moll et al. [20]. In brief, the antibody was pre-incubated with a synthetic 15-amino-acid peptide corresponding to the β-expansin antibody epitope (this particular peptide was used for the immunisation of a rabbit for the purpose of antibody production). Two different peptide concentrations were used for pre-incubation, resulting in a molar ratio of antibody to added peptide of 2:1 or 5:1. Specific labelling of β-expansins in Western blots was demonstrated by the absence of labelling when the antibody had been adsorbed with the peptide. *1D PAGE*, *1D Western blot and immune-detection*. For 1D Western blot, 15 μg proteins were extracted from the leaf surface by dipping the leaves in a mixture of chloroform:methanol (2:1; see method above) and were separated by 15% SDS-PAGE and transferred to a PVDF membrane as described below for the 1D Western blotting procedure. Before the PVDF membrane was incubated with the anti-ZmExpB antibody, the antibody was pre-incubated with the peptide as described above. Signals were detected by using chemi-luminescence as described for 2D Western blot.

### Data evaluation

For evaluating the pH data, the t-test as outlined by Köhler et al. [30] was calculated based on de-logarithmised pH values. Means are displayed as pH values. Epidermal cell size data were also evaluated by means of the t-test. In order to maintain an experiment-wise α of *p* < 0.05, multiple t-tests were adjusted according to the ‘Bonferroni-Holm’ method. Statistical significance is indicated by lowercase letters. LVDT curves are continuous kinetics of the leaf extension as affected by expansin treatment.

## Results

### Effects of salt stress on epidermal cell size

**Fig 1:**
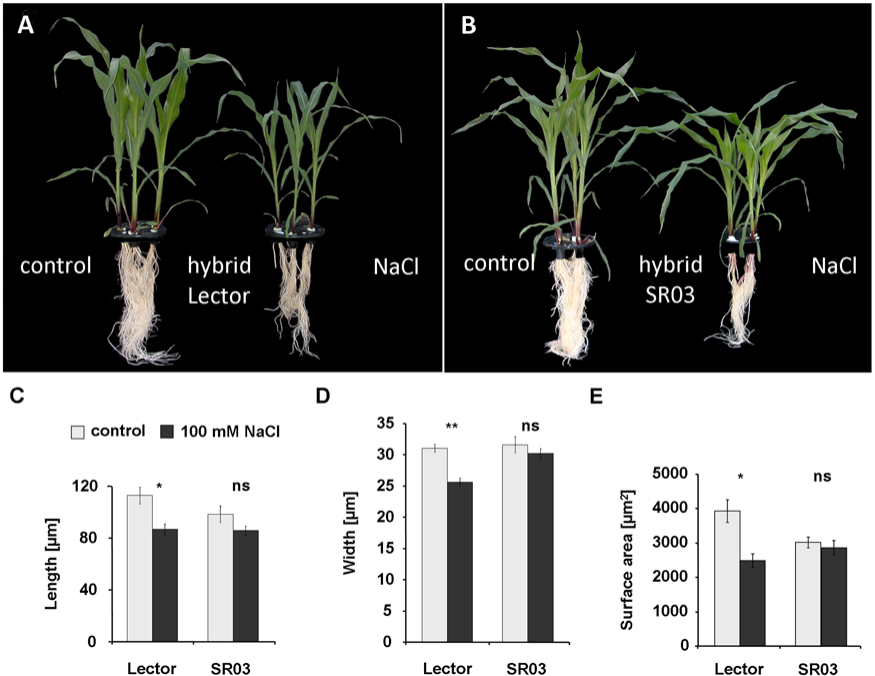
Epidermal cell size under condition of salt stress. Two different hybrids, namely the salt-sensitive Lector (A) and the relatively NaCl-resistant SR03 (B), were raised in the absence (left, control) and presence (right, 100 mM NaCl) of an 8-day NaCl stress treatment. The size of the epidermal cells was measured in the youngest expanding leaf in a region that had emerged from the sheaths. Epidermal cell length (A), epidermal cell width (B) and epidermal cell surface area (C) are shown. The data represent the means of four biological replicates (n = 4), each run in triplicate ± SE. Sizes of 12 cells per technical replicate were quantified. Asterisks indicate significant mean differences between salt treatments and controls (*p ≤ 0.05 and **p≤ 0.01; ns = not significant; t-test). Photo in (A) amended from Geilfus [37].

The phenotype of maize plants that were treated for 8 days with 100 mM NaCl is shown in Fig. 1. Compared with the control, salt stress led to a significant reduction in shoot and root development of the salt-sensitive variety Lector (Fig. 1A). This reduction was less pronounced in the more resistant variety SR03 (Fig. 1B). Subsequently, the dimensions of the epidermal cells were measured in the youngest expanding leaves in a region that had emerged from the sheaths. After 8 days of salt treatment, the length of the epidermal cells was significantly reduced in the salt-sensitive variety Lector, whereas no significant size reduction was measured in individuals of the salt-resistant hybrid SR03 (Fig. 1C). Similar results were obtained for cell widths (Fig. 1D) and cell surface area (Fig. 1E).

### Capacity of the epidermal cell wall to expand as modified by salinity

**Fig 2:**
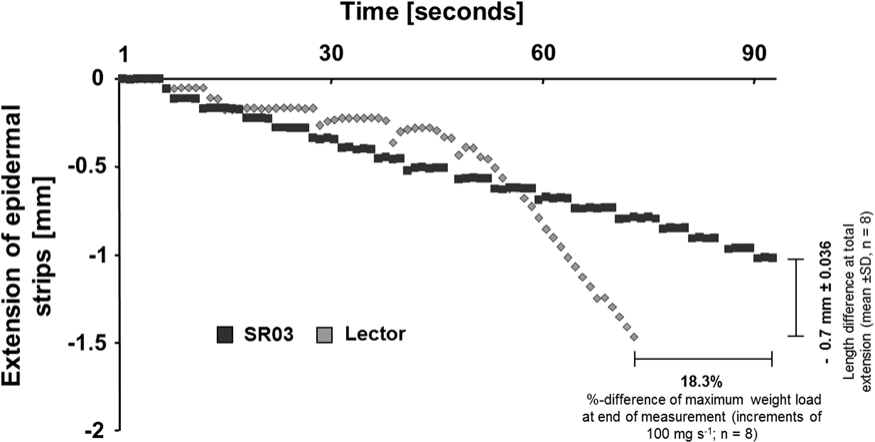
Capacity of the epidermal cell wall to expand. The capacity of the epidermal cell wall to expand was measured by using frozen-thawed epidermal strips of the youngest expanding leaf in a region that had emerged from the sheath. The maximal capacity of the epidermal cell walls to expand as measured with a linear variable differential transducer was plotted in millimetres over time. Light-grey, sensitive hybrid Lector; dark-grey, resistant hybrid SR03. Extension kinetics of salt-stress treatments were normalised by the corresponding control treatments. Representative kinetics of 8 equivalent recordings.

The capacity of the epidermal cell wall to expand was analysed by clamping epidermal strips in a linear variable differential transducer. The maximal capacity of the epidermal cell walls to expand under conditions of NaCl stress proved to be higher in the epidermis of the salt-resistant SR03 as compared with the epidermis taken from the salt-sensitive Lector (Fig. 2). Moreover, the epidermal strips of the salt-sensitive Lector tore earlier under tension as compared with the salt-resistant SR03, indicating a higher flexibility of the SR03 epidermis under NaCl stress. The kinetics of salt-stress treatments was normalised based on the corresponding control experiments (no NaCl).

### Ratiometric *in planta* measurements of leaf epidermal apoplastic pH

**Fig 3:**
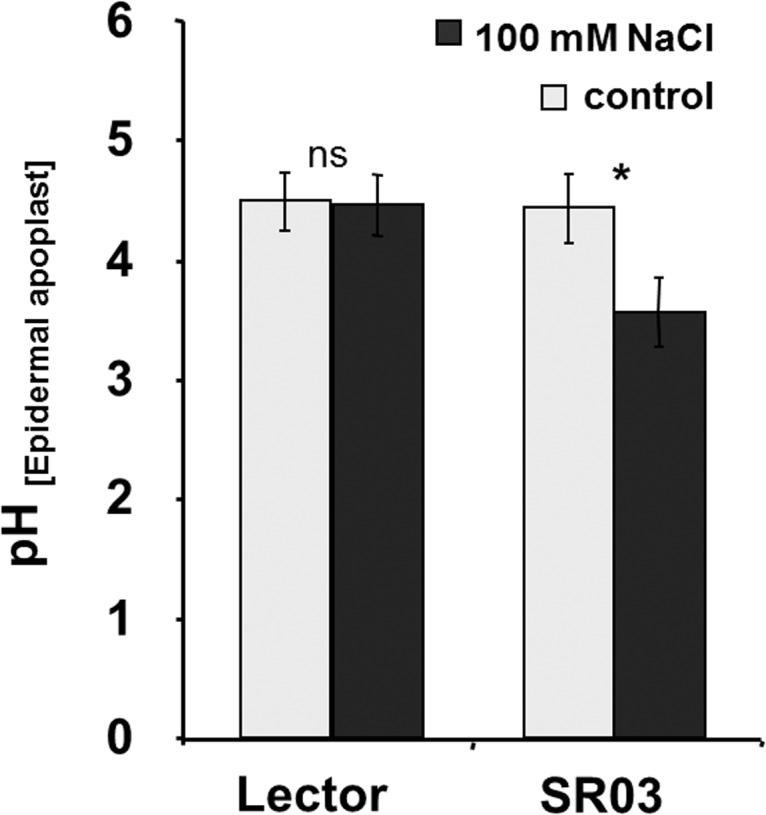
Effect of NaCl stress on the apoplastic pH in the leaf epidermal apoplast. *In planta* quantification of pH in the epidermal leaf apoplast in a region that had emerged from the sheaths. Light-grey: control; dark-grey: 100 mM NaCl, duration: 8 days. Lector: sensitive hybrid; SR03: resistant maize hybrid. Measurements were conducted on 45 different plants, with 5 regions of interest. The data represent means ± SE. Asterisks indicate significant mean differences between salt treatment and control with respect to the proton concentration (*p ≤ 0.001; ns = not significant; t-test).

Microscopy-based real-time ratio imaging was used for the *in planta* quantification of the pH in the apoplast bordered by epidermal cells. In the leaves of salt-sensitive Lector, the apoplastic pH was not changed upon the 8-d 100-mM NaCl treatment but was stable at a pH of ca. 4.5 (Fig. 3). In contrast, in response to the stress treatment, the pH was significantly acidified to ca. 3.7 in the relatively NaCl-resistant SR03. At a first glance, the low pH values seemed to be too acidic because, in an earlier publication that included the same SR03 maize variety, the pH was higher [31]. However, we improved the pH quantitation technique and were able to measure the pH non-invasively in an intact plant system; secondly, we used a pH-sensitive dye that enabled, because of the lower pKa, measurements in a more acid pH range.

### Protein abundance of β-expansin in the leaf surface proteome of Lector and SR03

**Fig 4:**
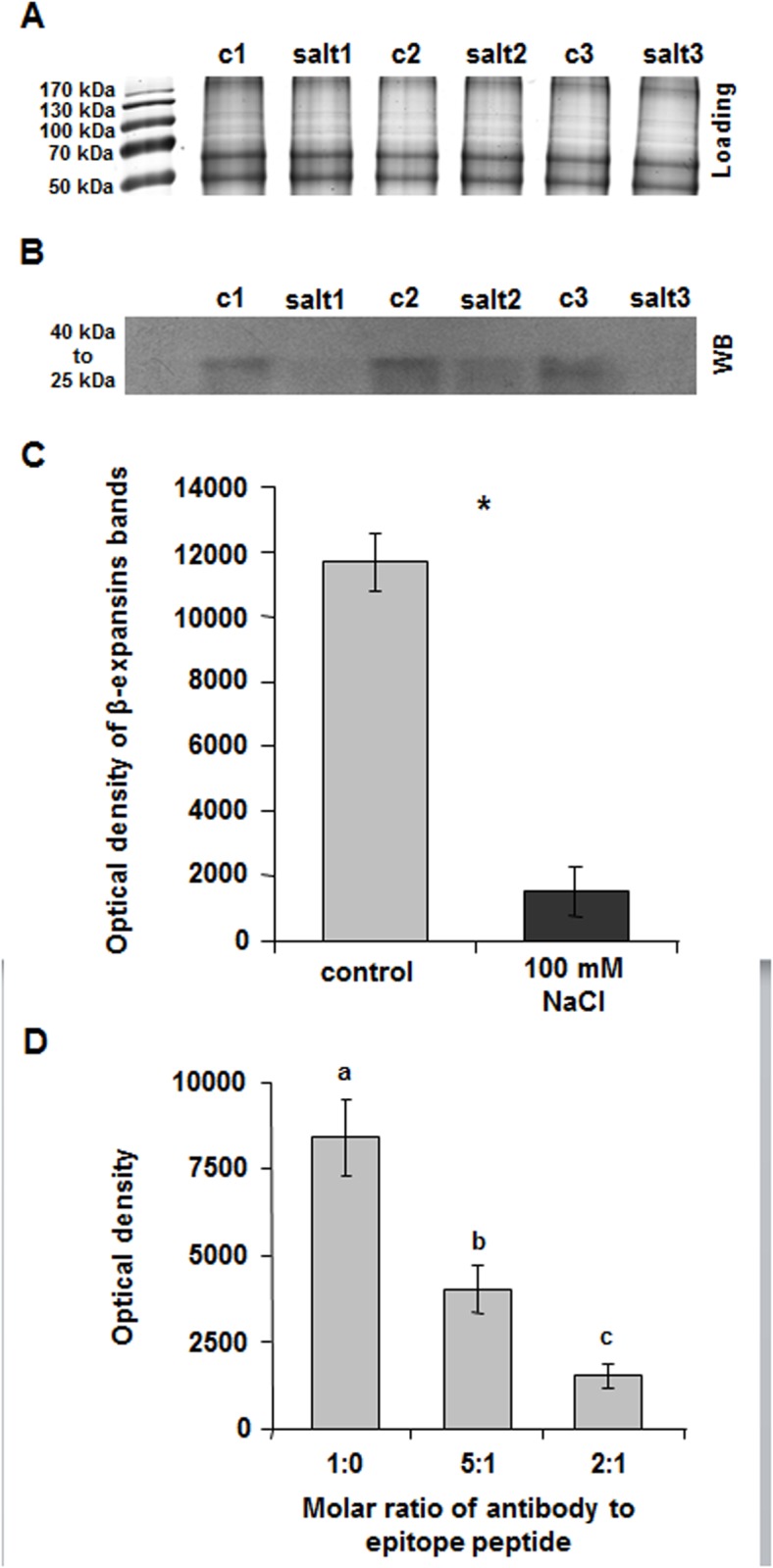
Western blot analysis of β-expansins in the epidermal cell wall of salt-sensitive Lector. (**A**) Coomassie-stained controls of the Western blot shown in (B) indicate equivalent protein levels in all lanes. c, control; salt, 8-d 100 mM NaCl treatment. Numbers 1–3 indicate biological replicates. (**B**) Western blot detection of β-expansins in expanding leaves of salt-treated (100 mM NaCl) and control plants. β-expansin bands appear between 40 and 25 kDa. c, control; salt, 8-d 100 mM NaCl treatment. Numbers 1–3 indicate biological replicates. (**C**) Densitometric analysis (TINA 2.08 software) of Western blot bands shown in (B). Bands were plotted as the average of the optical density (OD). Asterisks indicate significant difference (*p ≤ 0.01). (**D**) Specificity test of immunochemical β-expansin labelling. To exclude that unspecific signals were erroneously detected by the anti-peptide antibody (anti-ZmExpB), the antibody was pre-incubated with the epitope peptide before 1D Western blotting. A molar ratio of antibody to added peptide of 2:1 almost completely blocked WB labelling (signal intensity: 1513 density/mm^2^-background). A molar ratio of antibody to added peptide of 5:1 reduced the labelling (signal intensity: 4028 density/mm^2^-background). Under normal conditions without pre-incubation of the antibody with the epitope peptide (ratio of 1:0 = non-adsorbed antibody), the signal intensity was 8413 density/mm^2^-background.

**Fig 5:**
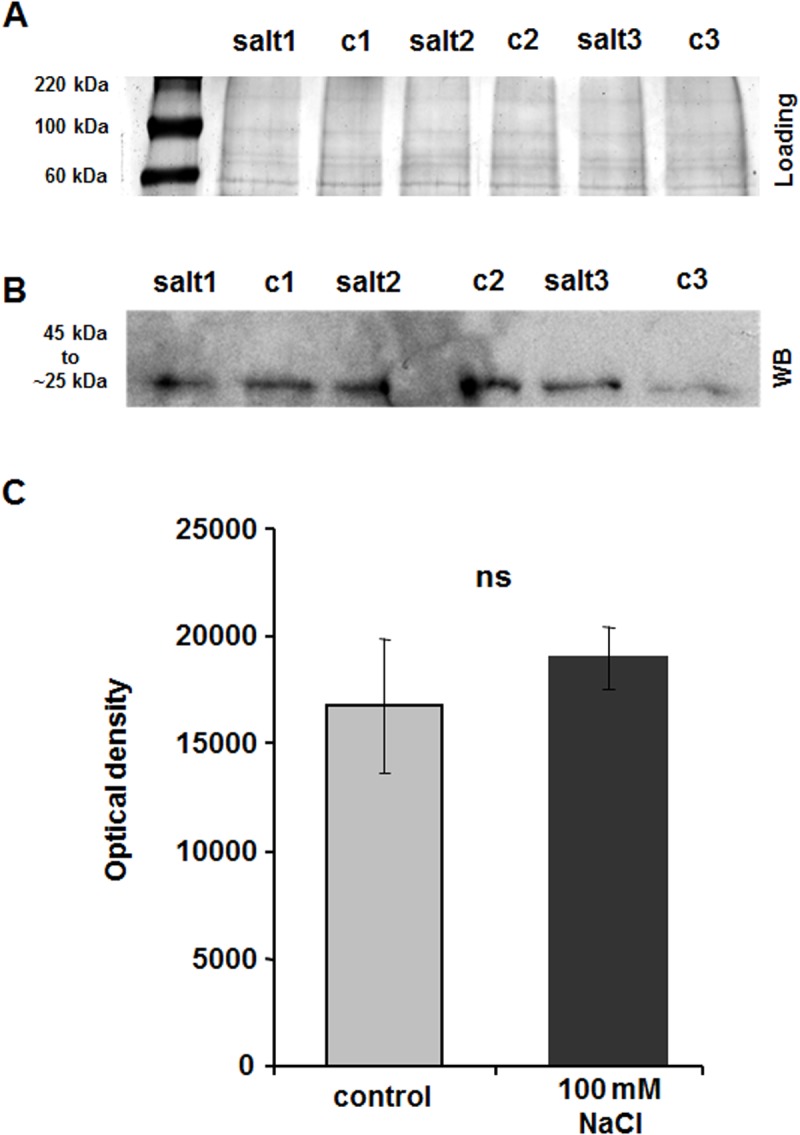
Western blot analysis of β-expansins in the epidermal cell wall of salt-resistant SR03. (**A**) Coomassie-stained controls of the Western blot shown in (B) indicate equivalent protein levels in all lanes. c, control; salt, 8-d 100 mM NaCl treatment. Numbers 1–3 indicate biological replicates. (**B**) Western blot detection of β-expansins in expanding leaves of salt-treated (100 mM NaCl) and control plants. β-expansin bands appear between 45 and 25 kDa. c, control; salt, 8-d 100 mM NaCl treatment. Numbers 1–3 indicate biological replicates. (**C**) Densitometric analysis of Western blot bands shown in (B). Bands were plotted as the average of the optical density (OD). ns = not significant.

The β-expansin proteins were isolated from the surface (*i*.*e*. outer epidermal wall) of expanding leaves from the salt-sensitive Lector (Fig. 4) and the more resistant SR03 (Fig. 5) and were separated via 1D-PAGE and subsequently labelled by using Western blot detection. The loading control of the PAGE gels documented equivalent levels of proteins in all lanes (Fig. 4A; Fig. 5A). In the case of the salt-sensitive Lector, Western blot analysis revealed the presence of a strong β-expansin signal band between 45 kDa and 25 kDa in the controls (Fig. 4B). In response to the salt treatment, the β-expansin protein bands were hardly detectable: densitometry analysis revealed a drastic reduction of the β-expansin protein abundance in the salt-sensitive Lector under salt stress (Fig. 4C). In contrast, β-expansin protein abundance was not affected in the more resistant SR03 (Fig. 5C). The specificity of the immunochemical β-expansin labelling was tested (Fig. 4D). The antibody was pre-incubated with the synthetic peptide that was used as the antibody epitope (see Material section). The pre-incubation of the antibody with the epitope peptide reduced or almost completely blocked specific labelling (ratio: 5:1 and ratio: 2:1, respectively). However, when non-adsorbed antibody was used (ratio: 1:0), the signal was most intense. These results establish the specificity of the anti-ZmExpB antibody for labelling β-expansins in maize.

## Discussion

Salt-sensitive crops such as maize react with strong growth reduction upon salt stress[9, 32–36]. The growth reduction is, among other reasons that are reviewed by Munns and Tester [6], attributable to a decline in cell wall mechanical properties. In maize leaves, Geilfus et al. [37] have found that the capacity of the wall to expand is severely reduced under conditions of an 8-d treatment with 100 mM NaCl.

A detailed literature search has revealed that the leaf epidermis is thought to form a rigid outer coat of the leaf and, by this means, contributes to the regulation of the expansion growth of leaves [13, 14]. However, with regard to the leaf growth reduction under salinity, the role of the epidermis in growth restriction has, to our knowledge, not yet been examined. Based on the assumption that epidermal tissue can restrict leaf growth by interacting with the inner cell layers [16, 38], we have investigated whether growth-related changes in the leave epidermis and its cell wall are correlated with the overall growth reduction of expanding maize leaves during an osmotic stress phase induced by salt treatment. This assumption is justified because studies on rice internodes have indicated that the epidermis can restrict plant growth through an inhibition of cell expansion [39, 40]. In accordance, Savaldi-Goldstein and Chory [14] have proposed that the cells in the epidermis can promote or restrict growth of the entire shoot by sending physical or chemical growth signals to the inner layers of the organ. Moreover, Marcotrigiano [38] reported about the large influence of the epidermis in controlling the size and rate of cell division in the mesophyll of leaves of developing tobacco plants.

We started our study by documenting the size of the epidermal cells that were located in a region that had recently (approx. 12 hours before) emerged from the sheaths. The salt-sensitive variety Lector (Fig. 1A) exhibited a significant reduction in the length (Fig. 1C), width (Fig. 1D) and surface area (Fig. 1E) of the epidermal cells upon the 8-d treatment with 100 mM NaCl. In contrast, SR03, which was characterised as being highly salt-resistant [17], maintained normal epidermal cell size under stressful conditions (Fig. 1C-E). Taking into account that the epidermis is considered to be able to restrict leaf expansion growth [12–14, 16, 41], we hypothesize that the growth-inhibited epidermal cells of the salt-sensitive variety (Fig. 1) contribute to a restriction in leaf growth, *i*.*e*. by its load-bearing physical properties directed against the turgor pressure from internal tissues[11]. The latter seems to be realistic, because the capacity of the epidermal cell walls to expand appears to be more reduced (Fig. 2) in the smaller epidermal cells (Fig. 1) of the salt-sensitive variety that exhibit strong leaf growth inhibition [36]. In accordance with this assumption, the wall stiffness of the relatively NaCl-resistant SR03 variety that maintained normal epidermal cell dimensions (Fig. 1) and leaf growth [36] under stress was less severely affected (Fig. 2)

The better capacity of the epidermal cell walls of the more resistant SR03 to expand (Fig. 2) might be attributable to the fact that the apoplast that surrounds the epidermal cells acidified under conditions of NaCl-induced stress (Fig. 3). This interpretation is in agreement with the acid-growth theory and also with the results of Van Volkenburgh and Boyer [42] who have found that higher growth rates in the leaves of maize (*Zea mays*) are associated with an acidification of the apoplastic space. Moreover, Fan and Neumann [43] have demonstrated that acidification of the root apoplast triggers growth in the elongations zones under conditions of water deficit. The apoplastic acidification detected in the resistant maize hybrid (Fig. 3) might be favourable for the activity of cell-wall-located expansin proteins in the epidermis that mediate acid-induced growth [15, 44–46]. In favour of this hypothesis, evidence has been presented that expansin transcripts and proteins are located in the epidermal tissue that acts as a 'growth-limiting cell layer' [16, 44].

This study revealed that in the case of salt sensitive maize, salt stress reduces the expression of β-expansin proteins that are located on the surface of the epidermal layer of growth-reduced leaves (Fig. 4). Taking into account the findings of Muller et al. [47] who associated expansin expression with growth processes that take place in the load-bearing epidermal tissue of maize leaves, we assume that the reduced epidermal β-expansin protein abundance (Fig. 4) contributes to a lower capacity of the epidermal cell walls to expand (Fig. 2). These tissue-specific effects are in accordance with previous findings that report about a reduced abundance of expansin transcripts and proteins in salt-stressed maize leaves [10, 36, 37]. However, the presented study expands these previous results from Geilfus et al. [36] since the current data now allow to discuss the specific role of the load-bearing epidermal tissue, and in particular its cell wall, in the context of the salinity-induced growth reduction. In contrast to the salt-sensitive Lector that reduced β-expansin protein abundance upon a 8-d treatment with 100 mM NaCl (Fig. 4), these treatment did not reduce the β-expansin protein abundance in the epidermal surface of the more resistant maize hybrid that maintained epidermal cell size (Fig. 5). This maintenance of the β-expansin protein abundance might also contribute to the better expansion capacity (Fig. 2) of the epidermal cell walls of the more resistant maize.

In summary, our data show a strong coherence between the salinity-induced cell wall stiffening and the size reduction of the epidermal cells in growth-reduced leaves of the salt sensitive maize hybrid. This reduced expansion capacity might be caused by a down-regulation of the growth-mediating β-expansins that are located in the epidermal cell walls. Of note, the data also demonstrate that the epidermal cell wall is more extensible in the more resistant maize hybrid that maintains its epidermal cell size and leaf growth under salinity. This correlates (1) with a shift of the epidermal apoplastic pH in a range that is more favourable for acid growth and (2) with maintenance of the β-expansin protein abundance in the epidermal cell walls.

**Fig 6:**
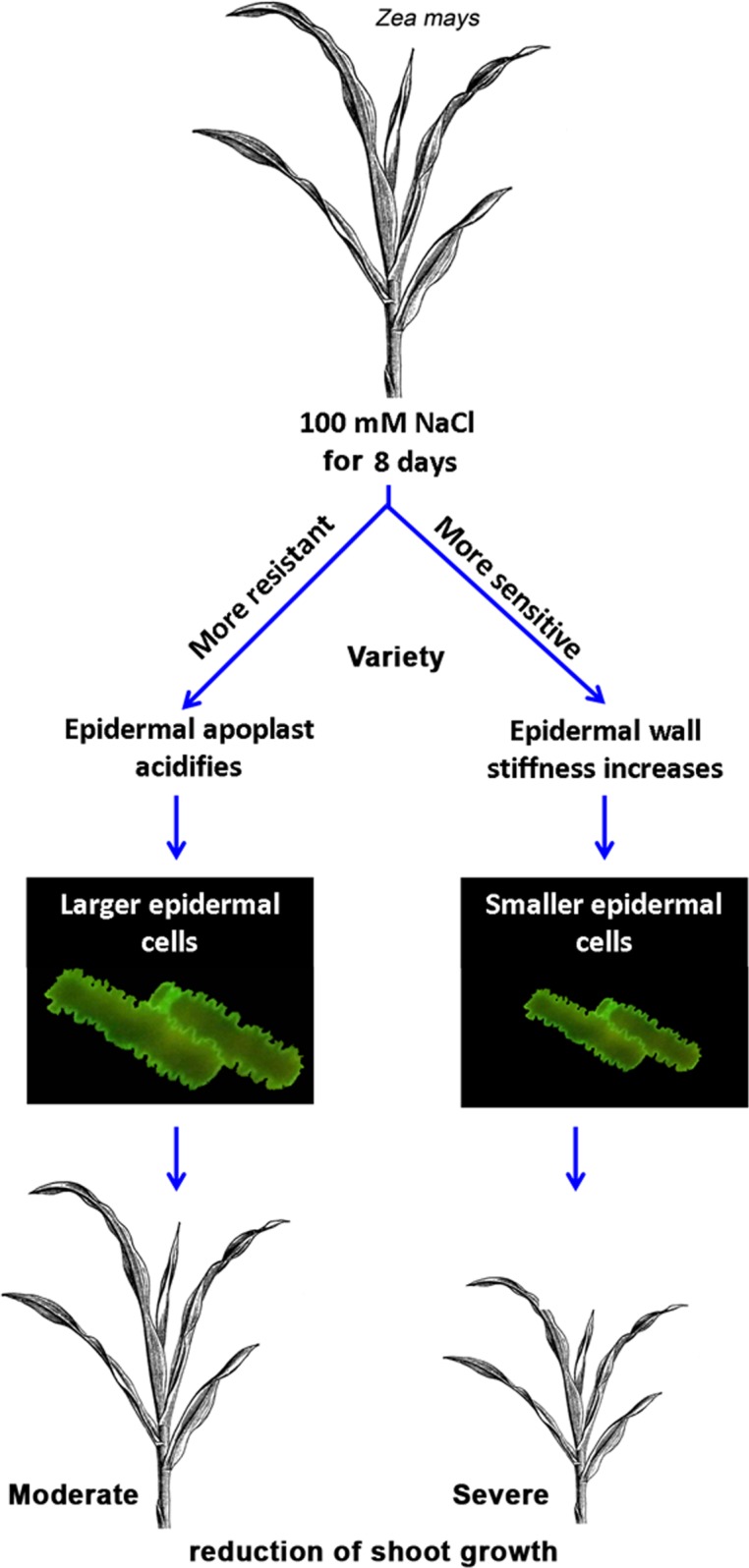
Model summarising the biophysical effects of NaCl stress on the leaf epidermis and its cell wall. Treatment of maize plants for 8 days with 100 mM NaCl stress causes a size reduction of the epidermal cells derived from growth-inhibited maize leaves. This might be attributable to the epidermal cell walls being stiffer because of the reduced abundance of cell-wall-loosening β-expansin proteins. Under stress, the more resistant maize variety acidifies its epidermal apoplast to a pH range that is more favourable for acid growth and the activation of wall-loosening expansin proteins. In good agreement with this observation, the more resistant variety has a better capacity for epidermal cell expansion. The salinity-induced cell wall rheological modifications of the epidermis emphasize a contribution of the load-bearing epidermis in restricting the expansion of the entire leaf, ultimately contributing to the salinity-induced growth reduction.

This approach disclosed physiological disturbances that have evolved in salt-sensitive maize upon confrontation with salinity, such as the stiffening of the growth-controlling epidermal layer. On the other hand, this study delivers valuable hints for understanding coordinated responses that create more favourable conditions for extension growth, such as the acidification of the epidermal apoplast in the more resistant maize variety (compare model in Fig. 6). Finally, salt stress appears to specifically impair cell wall rheological properties that control wall-loosening processes in size-reduced epidermal cells derived from growth-inhibited maize leaves. For this reasons, this study emphasizes the role of the epidermis as a growth restricting tissue that is involved in salinity-induced leaf growth inhibition.
